# Western diet leads to aging-related tumorigenesis via activation of the inflammatory, UPR, and EMT pathways

**DOI:** 10.1038/s41419-021-03929-9

**Published:** 2021-06-23

**Authors:** Ricardo Imbroisi Filho, Alan C. Ochioni, Amanda M. Esteves, João G. B. Leandro, Thainá M. Demaria, Mauro Sola-Penna, Patricia Zancan

**Affiliations:** 1 Laboratório de Oncobiologia Molecular, Departamento de Biotecnologia Farmacêutica, Faculdade de Farmácia, Universidade Federal Do Rio de Janeiro, Rio de Janeiro, 21941-902 Brazil; 2 Laboratório de Enzimologia e Controle do Metabolismo, Departamento de Biotecnologia Farmacêutica, Faculdade de Farmácia, Universidade Federal Do Rio de Janeiro, Rio de Janeiro, 21941-902 Brazil

**Keywords:** Melanoma, TOR signalling, Mechanisms of disease, Ageing

## Abstract

Among the principal causative factors for the development of complications related to aging is a diet rich in fats and sugars, also known as the Western diet. This diet advocates numerous changes that might increase the susceptibility to initiate cancer and/or to create a tissue microenvironment more conducive to the growth of malignant cells, thus favoring the progression of cancer and metastasis. Hypercaloric diets in general lead to oxidative stress generating reactive oxygen species and induce endoplasmic reticulum stress. Our results demonstrate that mice bearing tumors fed with a Western diet presented bigger tumor mass with increased insulin sensitivity in these tissues. Several markers of insulin signaling, such as AKT phosphorylation and mTOR pathway, are promoted in tumors of Western diet-fed animals. This process is associated with increased macrophage infiltration, activation of unfolded protein response pathway, and initiation of epithelial–mesenchymal transition (EMT) process in these tumor tissues. Summing up, we propose that the Western diet accelerates the aging-related processes favoring tumor development.

## Introduction

The incidence of most types of cancer increases dramatically with aging [1]. Undeniable epidemiological evidences also showed that overnutrition and unbalanced diets negatively influence morbidity and mortality in different types of cancer [2, 3]. The effects of diets interfering with cancer prognosis are, in part, due to diet-induced acceleration of cellular aging [4]. Indeed, different aspects of aging are accelerated by unbalanced diets, especially the so-called Western diet, rich in sugar and saturated fat, which promote cellular senescence and shortens longevity [5, 6]. Therefore, damaged cell accumulation including epigenetic changes, mitochondrial dysfunctions, cellular senescence, and activation of unfolded protein response (UPR) are characteristics of aging and cancer [7].

Cells in the tumor microenvironment also actively participate in the processes leading to tumor progression and metastasis, especially immune cells [8]. It has also been shown that aging is associated with an increase in low-grade systemic chronic inflammation called “inflammaging”, which contributes to cancer induction and progression [8]. However, less is known about the impact of cancer cells’ metabolic features on macrophages recruitment, survival, and activation in diet-induced aging. It is well established that macrophage activation (generating an inflammatory profile called M1-polarized macrophages) is present in a plethora of age-related pathologies such as insulin resistance, diabetes, obesity, and atherosclerosis [9–11]. However, during the tumorigenic process, macrophage activation plays an inverse role: the macrophage polarization to resolute M2-like profile favors tumorigenesis and tumor progression [12]. Studies have shown that M2 tumor-associated macrophages (TAMs), which have an immunosuppressive phenotype, are key drivers for tumor progression, especially in aging mice [13]. Moreover, stromal cells in the tumor microenvironment undergo metabolic changes, switching to a more glycolytic profile (aerobic glycolysis), and presenting mitochondrial dysfunction. As a result, the secretion of lactate, glutamine, and ketones, in addition to nitric oxide (NO) and reactive oxygen species (ROS) in the intratumoral milieu increases. These factors favor tumor development and aggressiveness [1] and markedly support the shift toward a mesenchymal phenotype, in a process referred to as epithelial–mesenchymal transition (EMT), which modifies the adhesion molecules expressed by the cancer cell, allowing it to adopt a migratory and invasive behavior [14].

Solid tumors are challenged by different conditions of the tumor microenvironment, such as hypoxia or lack of nutrients, due to poor vascularization that occurs with the rapid expansion of tumor mass. As a result, we observed a significant increase in endoplasmic reticulum (ER) stress with consequent activation of unfolded protein response (UPR) pathway, which attenuates the ER stress and promotes the survival and growth of tumor cells [15]. After ER stress induction, by ER protein-folding environment disruption, accumulated unfolded and misfolded proteins bind and sequester the chaperone immunoglobulin heavy-chain binding protein (BIP or GPR78), thus activating UPR. The UPR pathway comprises three parallel signaling branches: PRKR-like ER kinase (PERK), inositol-requiring protein 1α (IRE1α), and activating transcription factor 6α (ATF6α). Thus, UPR induces protein folding and transport, increases protein trafficking through the ER, ER-associated protein degradation (ERAD), and autophagy [15]. Therefore, UPR activation is required for oncogenic and malignant transformation.

This study aimed to evaluate the effects of a Western diet-based high-fat and high-sucrose (HFHS) diet on the tumor immune response in a mature adult mouse model. We have conducted a long-term (24 weeks) HFHS or chow diet study, and, subsequently, B16F10 orthotopic tumors were induced in immunocompetent mice to assess whether diet-induced insulin resistance or chronic inflammation triggers tumor progression. We identify overexpression of stearoyl-coenzyme desaturase (SCD1) as a molecular signature of HFHS tumors that largely predicts the tumor sensitivity to insulin by increasing insulin receptor (IR) expression and tumor growth. Moreover, our data showed that the HFHS diet induces cancer cell proliferation and EMT; for instance, it activates UPR in this mouse model.

## Materials and methods

### Animals and tumor-inducing

Twelve 10-week-old female mice (C57BL6/J) were individually housed under controlled temperature (23 °C) and 12/12 h daylight cycle (lights off at 18:00) with water and food ad libitum in the animal facility of the Faculty of Pharmacy, Federal University of Rio de Janeiro, Brazil. The sample size was calculated according to previously described [16]. The animal protocol used for the current work was performed according to what was previously approved by the Animal Care and Use Committee from the Health Sciences Center of the Federal University of Rio de Janeiro (CEUA/CCS/UFRJ 177/18). Mice were kept on a sterilized standard rodent diet (4.4% fat by kcal, total 3.6 kcal/g of diet, AIN-93M, Pragsoluções Biociências, Jaú, SP, Brazil) for 2 weeks to acclimate. At week 0 (12 weeks old), the mice were randomly divided into two groups (*n* = 6) that were either kept on the standard diet (chow) or transferred to an HFHS diet containing 65.4% fat and 19.6% carbohydrate (sucrose) by kcal for 26 weeks. The HFHS diet was prepared by Pragsoluções Biociências (Jaú, SP, Brazil) according to the directions described elsewhere [17]. Bodyweight gain and food intake were assessed three times a week. At week 23, B16F10 cells (10,000 cells in 50 μL phosphate-buffered saline) were subcutaneously injected into the back of the mice, forming a solid tumor with ~0.6 ± 0.2 cm^3^ after 10 days. At week 26, animals were anesthetized in chambers saturated with isoflurane and then sacrificed by cardiac puncture. On the day of sacrifice, all the mice were at the same age (36 weeks old) in both chow and HFHS groups. Fifteen minutes before sacrifice, animals were injected intraperitoneally with either saline (0.9% NaCl) or 2 U/kg insulin, in order to assess the insulin signaling in the tumors. Tumor samples were carefully collected and measured, where the volume was determined based on the caliper measurements, according to the described in the literature [18]. Following this, the tumors (and collected livers) were code-identified to guarantee blindness of the data analyses and then snap-frozen in liquid nitrogen. All the samples were included in data analyses and the whole study was conducted under the protocols approved by the animal care and handling committee of the Federal University of Rio de Janeiro.

### Cells

Mouse-derived skin melanoma cell line, B16F10, was obtained from the Cell Bank of Rio de Janeiro (www.bcrj.org.br, Duque de Caxias, RJ, Brazil) and was grown and maintained in Dulbecco’s modified Eagle’s medium with 25 mM glucose supplemented with 10% (vol/vol) heat-inactivated fetal bovine serum and 5 mM l-glutamine (Invitrogen, São Paulo, SP, Brazil) at 37 °C and 5% CO_2_ humidified incubator [19].

### Western blot

Analysis of total tumor necrosis factor-α (TNFα), phospho-nuclear factor-κB (NF-κB) (serine 536), total NF-κB, phospho-IκB (serine 32/36), phospho-Akt (threonine 308), phospho-Akt (serine 473), total Akt, phospho-ERK1/2 (threonine 202/tyrosine 204), total ERK1/2, total hypoxia-inducible factor-1α (HIF1α), total IRβ, total c-Myc, total PK-M2, total peroxisome proliferator-activated receptor gamma coactivator 1-alpha (PGC1α), total SCD1, phospho-PERK (threonine 981), total PERK, total ATF6, and total IRE1α was performed as previously described [20]. Immunoblotting was carried out using anti-Akt (dilution 1:1000, Cat# 9272, Cell Signaling Technology, Danvers, MA, USA), anti-phospho-Akt (S473) (dilution 1:1000, Cat# 9271, Cell Signaling Technology, Danvers, MA, USA), anti-phospho-Akt (T308) (dilution 1:1000, Cat# 9275, Cell Signaling Technology, Danvers, MA, USA), anti-ATF6 (dilution 1:1000, Cat# sc-22799, Santa Cruz Biotechnology, Santa Cruz, CA, USA), anti-c-Myc (dilution 1:1000, Cat# 5605, Cell Signaling Technology, Danvers, MA, USA), anti-ERK1/2 (dilution 1:1000, Cat# 4695, Cell Signaling Technology, Danvers, MA, USA), anti-phospho-ERK1/2 (T202/Y204) (dilution 1:1000, Cat# 4370, Cell Signaling Technology, Danvers, MA, USA), anti-HIF1α (dilution 1:500, Cat# NB100-449, Novus Biologicals, Centennial, CO, USA), anti-phospho-IκB (S32/36) (dilution 1:1000, Cat# 9246, Cell Signaling Technology, Danvers, MA, USA), anti-IRE1α (dilution 1:1000, Cat# 3294, Cell Signaling Technology, Danvers, MA, USA), anti-NF-κB (dilution 1:1000, Cat# ab31409, Abcam Plc, Cambridge, UK), anti-phospho-NF-κB (S536) (dilution 1:1000, Cat# 3036, Cell Signaling Technology, Danvers, MA, USA), anti-phospho-p70S6K (T421/S424) (dilution 1:1000, Cat# 9204, Cell Signaling Technology, Danvers, MA, USA), anti-p70S6K (dilution 1:1000, Cat# 9202, Cell Signaling Technology, Danvers, MA, USA), anti-PERK (dilution 1:1000, Cat# sc-13073, Santa Cruz Biotechnology, Santa Cruz, CA, USA), anti-phospho-PERK (T981) (dilution 1:1000, Cat# sc-32577, Santa Cruz Biotechnology, Santa Cruz, CA, USA), anti-PGC1α (dilution 1:1000, Cat# sc-5816, Santa Cruz Biotechnology, Santa Cruz, CA, USA), anti-PK-M2 (dilution 1:1000, Cat# 4053, Cell Signaling Technology, Danvers, MA, USA), anti-IRβ (dilution 1:500, Cat# sc-711, Santa Cruz Biotechnology, Santa Cruz, CA, USA), anti-SCD1 (dilution 1:1000, Cat# 2794, Cell Signaling Technology, Danvers, MA, USA), and anti-TNFα (dilution 1:1000, Cat# ab1793, Abcam Plc, Cambridge, UK). Anti-β-actin (dilution 1:1000, Cat# 4967, Cell Signaling Technology, Danvers, MA, USA) and anti-eEF2 (dilution 1:1000, Cat# 2332, Cell Signaling Technology, Danvers, MA, USA) were used as the loading control. Horseradish peroxidase-conjugated anti-mouse and anti-rabbit secondary antibodies were from Jackson ImmunoResearch Laboratories (West Grove, PA, USA). Immunoreactive bands were revealed by Amersham ECL Western Blotting Reagent (Cat# RPN2124, GE Healthcare Bio-Sciences, Pittsburg, PA, USA) and scanned using C-DiGit Blot Scanner (LiCor, Lincoln, NE, USA). Densitometry was quantified using the ImageJ software (National Institutes of Health, Bethesda, MD, USA).

### Quantitative real-time PCR

Freeze-powdered tumor and liver samples were homogenized in 0.5 mL of TRIzol reagent (Thermo Fisher Scientific, Carlsbad, CA, USA) and total RNA was purified following the manufacturer’s indication. Total RNA was used for complementary DNA (cDNA) synthesis using a High-Capacity cDNA Reverse Transcription Kit (Thermo Fisher Scientific, Carlsbad, CA, USA). After RNA reverse transcription, quantitative real-time PCR (qPCR) was performed using SYBR Green GoTaq Gene Expression Kit (Cat. #A6002, Promega, Fitchburg, WI, USA) or with commercially available TaqMan primers and probe sets and TaqMan Universal Master Mix II (Cat. #4426710, Applied Biosystems, Foster City, CA, USA). The sequences of custom-made oligos for gene expression assays are as follows: *Adgre1* (Fw: CCGTCAGGTACGGGATGAAT; Rv: AGAAGTCTGGGAATGGGAGC), angiopoietin-2 (*Angpt2*) (Fw: TCAACAACTCGCTCCTTCAGA; Rv: AGCTCTTGGAGTTGGGTGATG), *Arg1* (Fw: CCTCGAGGAGGGGTAGAGAAA; Rv: GGTCTCTCACGTCATACTCTGTTT), *Atf4* (Fw: ACATTCTTGCAGCCTTTCCC; Rv: TAAGCAGCAGAGTCAGGCTT), *Bip* (Fw: GGTGCAGCAGGACATCAAGTT; Rv: CCCACCTCCAATATCAACTTGA), *Cdh2* (Fw: ATGGCCTTTCAAACACAGCC; Rv: CTCCGTAGAAAGTCATGGCAG), *Chop* (Fw: CTGCCTTTCACCTTGGAGAC; Rv: CGTTTCCTGGGGATGAGATA), *Egr2* (Fw: GTGGCGGGAGATGGCATGAT; Rv: TCGGATACGGGAGATCCAGG), *Fizz1* (Fw: AACTTCTTGCCAATCCAGCTAAC; Rv: GGAGGCCCATCTGTTCATAGT), *Fpr2* (Fw: CCAGTGATTCAAGCACCAGTT; Rv: TCACAGACTTCATGGGGCCTT), matrix metallopeptidase 9 (*Mmp9*) (Fw: GCGTCATTCGCGTGGATAAG; Rv: AGGCTTTGTCTTGGTACTGGA), *Sirt1* (Fw: GCGGCTTGAGGGTAATCAAT; Rv: GACAAGACGTCATCTTCAGAGTC), *Sirt3* (Fw: AACGTGGCAAGCTGGATGG; Rv: GATCTGCTGTGTGACTTCCCC), *Snail* (Fw: TGGTCAGGAAGCCGTCCG; Rv: CTGGAAGGTGAACTCCACACAC), telomerase reverse transcriptase (*Tert*) (Fw: TGCAGTCTGTGTTTCGGAGA; Rv: CGTAAAAGCAACCCATCCCG), transforming growth factor β (*Tgfb1*) (Fw: TACCAACTATTGCTTCAGCTCCAC; Rv: CCAACCCAGGTCCTTCCTAAA), *Twist1* (Fw: TCAGCTACGCCTTCTCCGTC; Rv: ATGACATCTAGGTCTCCGGCCT), *Vim* (Fw: TCCTCTGGTTGACACCCACT; Rv: TGATCACCTGTCCATCTCTGGT), spliced form of X-box binding protein 1 (*Xbp1s*) (Fw: GAGTCCGCAGCAGGTG; Rv: GTGTCAGAGTCCATGGGA), and *Xbp1t* (Fw: AAGAACACGCTTGGGAATGG; Rv: ACTCCCCTTGGCCTCCAC). All the sequences were designed using Primer-BLAST tool [21] and all qPCR conditions were optimized by following international standards [22], using a fixed amplification program of 2 min at 95 °C, 40 cycles of 15 s at 95 °C, and 1 min at 60 °C, followed by a dissociation curve to assure the amplification of a single amplicon. Relative messenger RNA (mRNA) expression was calculated by the 2^−ΔCt^ method (10.1016/j.biopha.2017.01.091), using *Actb* (Fw: TGAACCCTAAGGCCAACC; Rv: CACAATGCCTGTGGTACG) as the reference gene. The amplification efficiency for all used primers was in the range of 95–105%. For TaqMan gene expression assays, the following oligos were used: Il1b (Mm00434228_m1) and TNFα (Mm00443258_m1), and the mRNA expression was calculated by the 2^−ΔCt^ method, using *Hprt* (Mm03024075_m1) as the reference gene.

### Statistical analysis

Data are expressed as mean ± standard error of the mean (SEM). Two-way analysis of variance followed by Tukey’s test, Mann–Whitney test, or unpaired Student’s *t* test was used as appropriate to compare groups, as stated in the figure legend (GraphPad Prism, v. 9.00, La Jolla, CA, USA). All results were considered statistically significant at *P* < 0.05. All statistics were performed meeting the assumptions of the tests, and estimation of variance was considered for each test.

## Results and discussion

### Immune response in B16F10 tumors

**Fig. 1:**
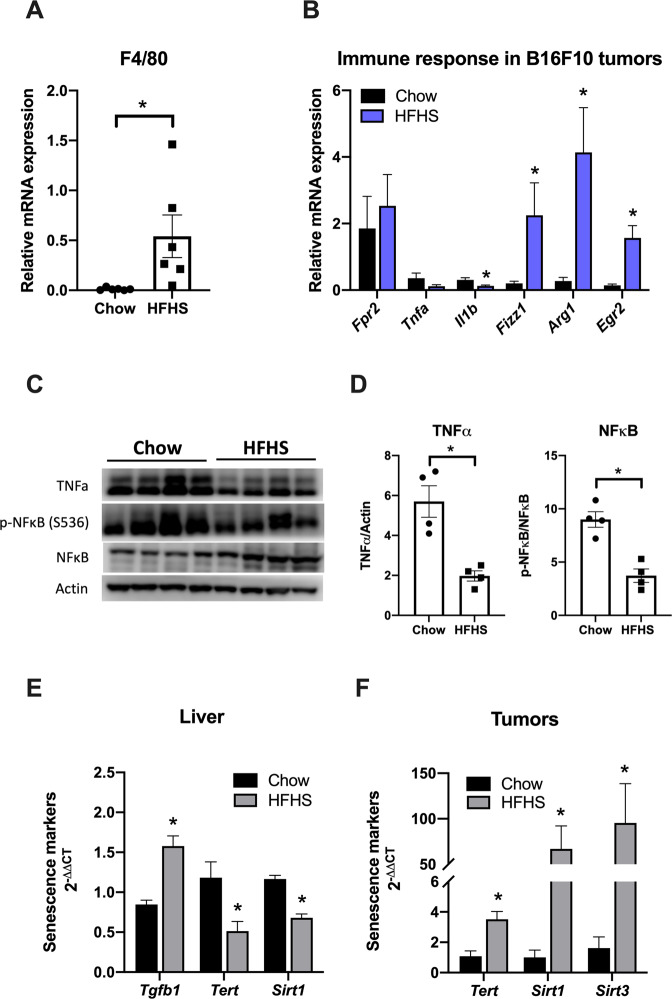
Macrophage infiltration, M2-like polarization, and anti-inflammatory response increase in tumors of HFHS-fed mice. C57BL/6J mice were treated with chow or HFHS diet as described in “Materials and methods” section. Then, B16F10 cells (10,000 cells in 50 μL PBS) were implanted subcutaneously in the back of wild-type mice. After 3 weeks, mice were sacrificed and tumor tissues were subject to several analyses. mRNA levels of macrophage infiltration (F4/80 or *Adgre1*, **A**) and immune response (*Fpr2*, *Tnfa*, *IL1b*, *Fizz1*, *Arg1*, and *Egr2*, **B**) from tumor tissues were measured by qPCR analysis (*n* = 6). *P < 0.05 as compared to control (Mann–Whitney test). **C** Western blot analysis of proteins associated with tumor’ inflammatory response to HFHS diet (each lane in the Western blot represents a tumor from a different animal). **D** TNFα, pNF-κB (S536), and NF-κB were quantified using the ImageJ software and the quantification of each staining is plotted in the panels normalized by the quantification of the total protein staining. β-Actin was used as the loading control. Values are mean ± SEM of four independent animals (*n* = 4). *P < 0.05 as compared to control (unpaired Student’s *t* test). **E** Relative mRNA expression of *Tgfb1*, *Tert*, and *Sirt1* from mice livers measured by qPCR analysis. **F** Relative mRNA expression of *Tert*, *Sirt1*, and *Sirt3* from tumors measured by qPCR analysis (bars are mean 2^−ΔΔCt^ ± SEM; *n* = 6; *P < 0.05, Mann–Whitney non-parametric test as compared to control).

To determine the effect of long-term HFHS diet on tumor immune response, macrophage infiltration and polarization phenotype were assessed by evaluating the expression of specific markers in tumors from C57Bl6/6J mice fed with chow or HFHS diet. Specific mouse macrophage surface marker F4/80 was overexpressed (38-fold) in the tumors from HFHS diet-fed animals, as compared to tumors from chow-fed animals (Fig. 1A). F4/80 has been extensively used to detect macrophages in different tissues from mice, including tumors, since F4/80 is also a marker of TAMs surface [23]. It is well established that obesity favors systemic inflammation, adipokine dysregulation, insulin resistance, dysbiosis, and changes in the immune system [5]. In fact, aging and obesity are regulated by similar pathways and, thus, studies have shown that obesity accelerates the aging process and promotes telomere shortening [4]. Thus, our data here strongly indicates an increased macrophage infiltration in the tumors from HFHS diet-fed animals.

Aimed to assess the polarization profile of macrophages infiltrating HFHS diet-fed animals, we assessed the expression of M1-polarized macrophage-specific markers (*Fpr2*, *Tnfa*, and *Il1b*) and M2-polarized macrophages markers (*Fizz1*, *Arg1*, and *Egr2*). TAMs are also known to express M2-like surface markers, such as the above-mentioned markers, among other markers, presenting an immunosuppressive effect [24]. In this study, we observed the upregulation of *Fizz1*, *Arg1*, and *Egr2* (Fig. 1B), confirming that the HFHS diet not only increases macrophage infiltration in tumor tissue but also contributes to their polarization to the M2-like phenotype, characterizing TAM infiltration. By contrast, genes associated with pro-inflammatory immune activation were either unaffected (*Fpr2*) or downregulated (*Tnfa* and *Il1b*) in tumors from HFHS diet-fed animals (Fig. 1B). These data indicate that animals fed with HFHS diet show reduced infiltration of M1-polarized macrophages.

It is described that reduction in the expression of M2-associated genes and polarization of macrophages to pro-inflammatory profile (M1) in tumors is associated with increased survival of patients with various forms of cancer [24]. This is quite a contrast to what we observed here, suggesting that HFHS-fed animals would exhibit a poor prognosis. Moreover, TNFα, which is downregulated in tumors from HFHS diet-fed animals, is known to be particularly deleterious to tumor development [24]. Here, we confirm the downregulation of this cytokine by evaluating its protein levels, and phosphorylation of its downstream targets, IκB and NF-κB, decreased in HFHS diet-fed animals (Fig. 1C–F). These results suggest that, unlike the M1 polarization that is usually observed in metabolic tissues [25], a Western-style diet promotes M2 polarization of macrophages residing in murine tumors, which could further contribute to the tumor progression.

Senescence-associated secretory phenotype has crucial roles in promoting obesity-associated cancer development in mice [26]. Here, we confirmed that HFHS diet accelerates telomere shortening by increasing *Tgfb1* gene expression and, consequently, reducing the expression of *Tert* (Fig. 1E). In addition, the aging process of these mice is supported by the lower expression of SIRT1 in the livers of Western diet-fed animals (Fig. 1E). However, as expected, tumor senescence was not observed. Our results indicate that the telomerase reactivation process is activated in the tumors of HFHS mice through higher *Tert* expression (~3.5 times higher than in tumors of the chow group) (Fig. 1F). Consistently, SIRT1 and SIRT3 expressions are also significantly increased in these tumors (Fig. 1F).

### Effect of HFHS on body and tumor weight and signaling parameters

**Fig. 2:**
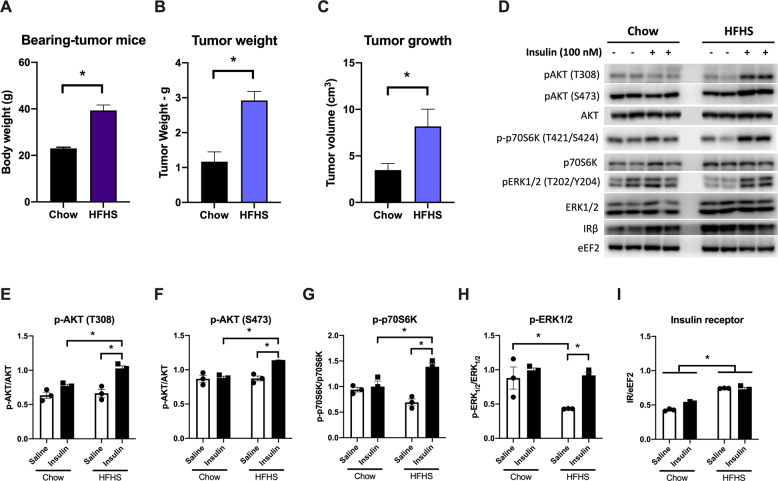
HFHS diet significantly increases tumor weight and volume and mice body weight. Twelve-week-old C57Bl6/6J female mice were fed standard chow or HFHS diet for 26 weeks. Then, B16F10 cells (10,000 cells in 50 μL PBS) were implanted subcutaneously in the back of wild-type mice. After 3 weeks, mice were killed and tumor tissues were subject to several analyses. **A** Body weight of the mice at the end of the experiment (g ± SEM). **B** Tumor weight (g ± SEM, *n* = 6). **C** Tumor volume measured with digital calipers (cm^3^ ± SEM). **D** Insulin signaling was analyzed by Western blots in protein lysates of the tumors from chow and HFHS-fed mice, which were treated with an insulin bolus after having food withheld for 6 h. Representative Western blot of total and phosphorylated AKT, p70S6K and ERK1/2, and insulin receptor β (IRβ) as shown for chow (left) and HFHS (right) group. **E**–**I** pAKT (T308)/AKT, pAKT (S473)/AKT, p-p70S6K (T421/S424)/p70S6K, pERK1/2 (T202/Y204)/ERK1/2, and IRβ/eEF2 were quantified using the ImageJ software, and the quantification of each staining is plotted in the panels normalized by the quantification of the total protein staining. eEF2 was used as the loading control. Values are mean ± SEM of three independent animals (*n* = 3). *P < 0.05 as compared to control (unpaired Student’s *t* test for (**A**–**C**), and two-way ANOVA, followed by Tukey’s post test for **E**–**I**).

Western diets are better known for their effects on body composition and insulin resistance [27]. Indeed, animals fed with HFHS diet presented approximately twice the body weight by the end of the protocol compared to the chow group (Fig. 2A). Analogously, tumors from HFHS diet-fed mice were relatively heavier (2.92 ± 0.26 and 1.17 ± 0.28 g, respectively) and larger (8.17 ± 1.84 and 3.48 ± 0.70 cm^3^, respectively) than tumors from standard chow-fed mice (Fig. 2B,C). These results are consistent with the view provided by Zitvogel et al. [28] about the relationship between nutrition, inflammation, and cancer. It is clear that nutrition has considerable effects on the incidence and progression of cancer by Western-style diet, associated with a sedentary lifestyle, positively modulates tissue inflammation, and decreases the systemic immunosurveillance [28].

Surplus calories provide an abundance of energy-rich metabolites (such as glucose, amino acids, and fatty acids) in addition to trophic factors (such as insulin, leptin, and insulin-like growth factor), which stimulate the proliferation of tumor cells. In addition to its effects on cell metabolism, insulin has an important mitogenic effect, fostering cell proliferation in both normal and malignant cells [29]. However, serum insulin levels do not influence cancer progression due to constitutive activation of the insulin signaling pathway observed in tumor cells [30]. Therefore, with the aim to clarify the HFHS diet effects on tumor growth signaling, we probed tumor responsiveness to insulin. Insulin signaling action was analyzed by assessing the phosphorylation of key proteins of this pathway, such as AKT, p70S6K, and ERK1/2. Our data indicated a constitutive activation of the insulin signaling pathway in tumor cells as seen in chow group tumors. This conclusion is due to the lack of insulin-stimulated phosphorylation of AKT both at Thr308 and Ser473 residues (Fig. 2D (left panel), E, F), p70S6K at Thr421/Ser424 (panel 2G), and ERK1/2 at Thr202/Tyr204 (panel 2H). In contrast, tumors from HFHS-fed mice responded to exogenous insulin administration, presenting a significant increase in insulin signaling pathway-related protein phosphorylation (Fig. 2D (right panel), E–H). Therefore, we identified that a Western-style diet leads to a disruption of the constitutive insulin signaling in tumor tissues, which will be discussed below. Accordingly, this increase in insulin responsiveness of the HFHS-fed mice tumors can lead to increased insulin-mediated mitogenesis that will be responsible for the enhanced tumor growth as compared to the tumor size seen in chow-fed mice (Fig. 2A, B).

A number of studies have indicated that obesity is also associated with enhanced cancer mortality [31, 32] and, according to research evidences, this is related to insulin-sensitive cancer cells, which are positive to IR in immunohistochemistry [33, 34]. As shown in Fig. 2D,I, tumors from animals fed with HFHS diet present enhanced IR content, which might contribute to the increase of the cancer cells’ sensitivity to insulin signaling and mitogenic effects. Interestingly, IR is predominantly expressed in tumor cells but not in stromal adipocytes or inflammatory cells in tumors [34]. Other researchers have also demonstrated that inactivation of tumor suppressor p53 promotes IR overexpression in cancer cells [35, 36], which can be a similar mechanism currently observed. Moreover, other studies have already shown that overexpression of IR in cancer cells may explain their greater sensitivity to hyperinsulinemia [37].

It is very important to keep in mind that, in this work, we are not dealing with tissues known for their responsiveness to insulin, such as adipose tissue or the liver. In these tissues, the Western diet is associated with the epidemic spread of obesity and type 2 diabetes mellitus (T2DM) and consequent chronic subclinical inflammation and metabolic syndrome. In this current study, we are addressing the effects of the diet on tumor development, since numerous epidemiological studies have demonstrated that both obesity and T2DM are important risk factors for a variety of malignancies [38, 39] and that hyperinsulinemia is a major cancer risk factor of obese and diabetic patients [40]. In addition, Belfiore and Malguarnera described in 2011 that circulating insulin may affect cancer growth because insulin resistance is normally restricted to the metabolic pathway, whereas IR signaling pathways leading to cell proliferation and migration are unaffected and even enhanced [37]. Moreover, malignant cells often overexpress IRs that may reach or exceed expression levels physiologically observed in classical insulin target organs, such as liver or adipose tissue [41, 42]. This evidence confirms the data revealed in this study pointing to a positive effect of hyperinsulinemia on the development of tumors.

### HFHS diet induces EMT through activation of SCD1

Since insulin signaling is also related to EMT, we investigated whether insulin-sensitive HFHS tumors present a cellular phenotype transition to a more aggressive profile. EMT is a process in which epithelial cells that have a strong cell–cell connection and apicobasal polarity acquire mesenchymal features. As a consequence, apicobasal polarity is lost, cell cytoskeleton is reorganized, intercellular interactions are reduced or eliminated and cancer cells gain motility by losing anchorage. This process explains how cancer cells can invade adjacent tissues, generate metastases, and resist cell death and chemotherapy [14, 43]. The transcription factors involved in EMT are those of the ZEB family (zinc finger and homeodomain), such as SNAIL (zinc-finger protein SNAI1) and TWIST (Twist-related protein 1). All of them work by decreasing the expression of genes related to epithelial characteristics and increasing the expression of genes involved in mesenchymal characteristics [14, 44–47].

**Fig. 3:**
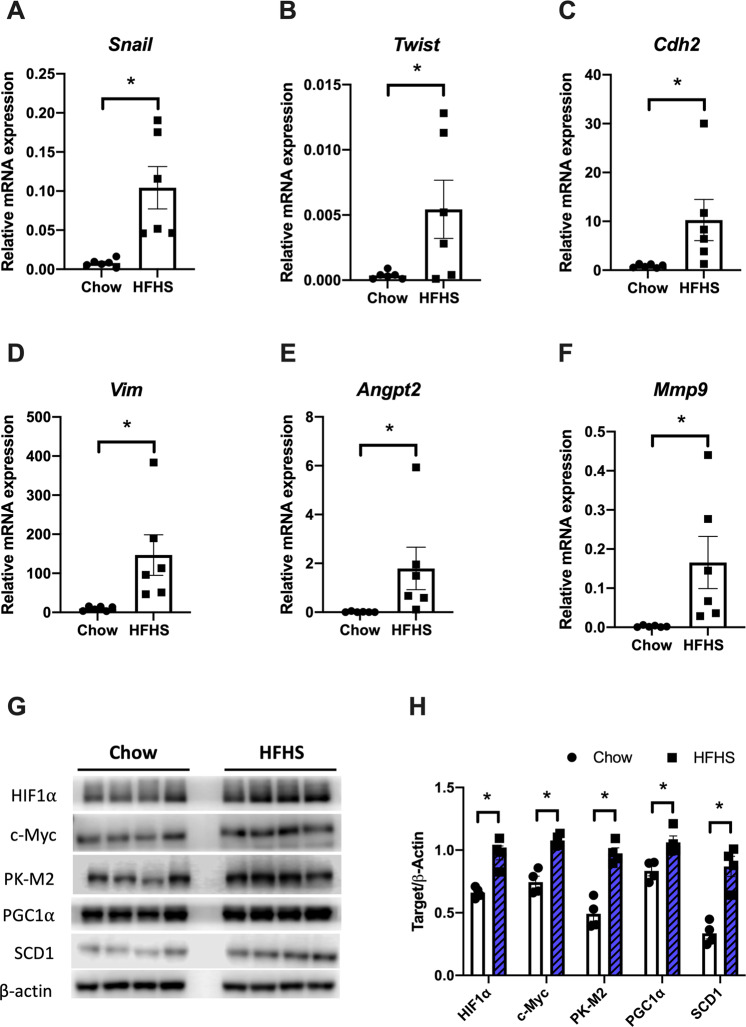
HFHS diet induces EMT, angiogenesis, and aggressiveness through oncogenic stimulation. mRNA expression of EMT-related genes (*Snail*, *Twist*, *Cdh2*, and *Vim*, **A**–**D**), angiogenesis (*Angpt2*, **E**), and matrix metalloproteinase 9 (*Mmp9*, **F**) were assessed by qPCR (*n* = 6). **G** Western blot of protein extract from tumor tissues of mice fed chow or HFHS diet. **H** Densitometric quantification of Western blots. Results are expressed as means ± SEM (*n* = 4). *P < 0.05 as compared to control (Mann–Whitney test for (**A**–**F**) and unpaired Student’s *t* test for **H**).

Our results showed that the HFHS diet upregulates several EMT markers such as *Snail*, *Twist*, *N-Cadherin*, and *Vimentin* in tumors from the animals (Fig. 3A–D). In addition, the metastatic potential of these tumors is also enhanced, as suggested by augmented mRNA levels of *Angpt2* (Fig. 3E) and *Mmp9* (Fig. 3F). It is well established that during aging, systemic angiogenesis tends to decelerate. However, the tumor microenvironment overcomes this deficit by increasing secretion of angiogenic factors, such as vascular endothelial growth factors (VEGFs), and expression of pro-angiogenic factors, such as *Angpt2* [48]. Moreover, other studies have indicated the importance of metalloproteinases, such as MMP9 and other proteases, as drivers of tumorigenesis [49, 50].

EMT observed in tumors from HFHS diet-fed animals is correlated with overexpression of SCD1 protein (2.5 times as compared to the chow group; Fig. 3G, H). Overexpression of SCD1 is positively associated with melanoma progression [51], and SCD1 is highly functional in cancer cells [52]. Indeed, SCD1 expression is stimulated by HIF1α and c-Myc [53], which are also overexpressed in tumors from HFHS diet-fed animals (Fig. 3G, H). These oncogenes promote the expression of many tumorigenic markers, including SCD1 and the M2 isoform of pyruvate kinase (PK-M2), which is also upregulated in these tumors (Fig. 3G, H). Moreover, HIF1-mediated upregulation of SCD1 results in the activation of Akt (here presented in Fig. 2) and SREBP-1 [53]. In addition, we observed a 1.3-fold increase in PGC1α expression, which contributes to SCD1 overexpression, since PGC1α binds to the SCD1 promoter and augments SCD1 gene expression [52]. Together, these data indicate that the stimulus by mitogenic factors in HFHS-fed mice tumors promotes upregulation of SCD1 expression and, consequently, activation of pro-growth signaling cascades observed in Fig. 2 (ERK1/2, Akt, and mTOR/p70S6K). Our results are reinforced by other studies, which specify that SCD1 expression is positively associated with patient age and cancer aggressiveness [54], highlighting the relationship between SCD1 expression and the rate of cell proliferation [55].

### Nutrient status induces UPR in HFHS-fed mice tumors

**Fig. 4:**
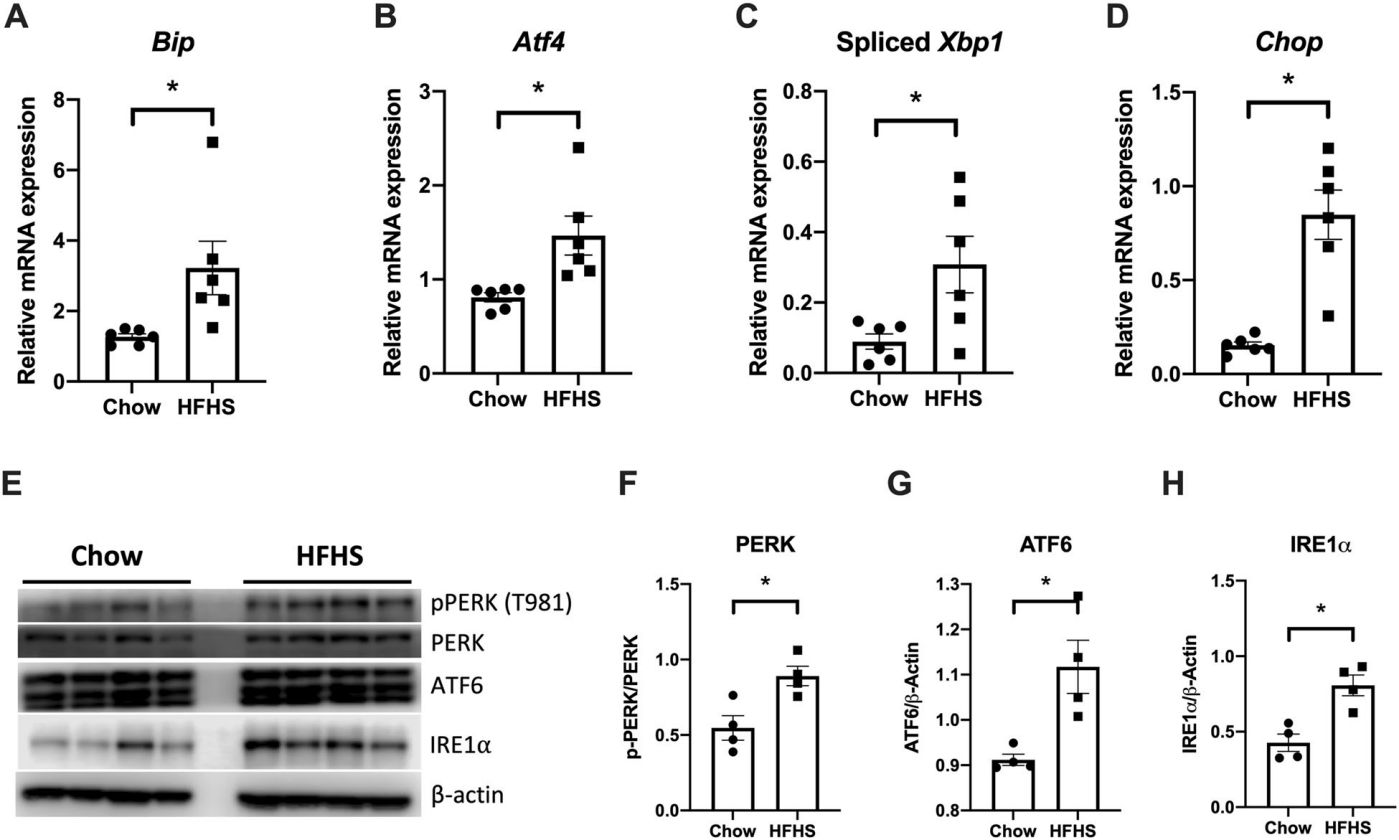
ER stress and UPR activation by HFHS died in tumor tissues. **A–D** mRNA expression of *Bip*, *Atf4*, spliced *Xbp1*, and *Chop* genes were assessed by qPCR. Results are expressed as means ± SEM (*n* = 6). **E** Western blot analysis of proteins associated with the UPR signaling pathway in tumors from chow or HFHS-fed mice. Representative Western blot of PERK, IRE1α, and ATF6 are shown for chow (left panel) and HFHS (right panel) groups. **F**–**H** β-Actin was used as the loading control. Values are mean ± SEM of four independent animals (*n* = 4). All plotted values are statistically different from the chow group (Mann–Whitney test for panels **A**–**D** and unpaired Student’s *t* test for panels **F**–**H**).

ER stress and UPR activation are documented in the development of diverse cancer types having important roles in every aspect of cancer development [15]. In macrophages, ER stress promotes the M2 macrophage phenotype that supports tumor growth [56]. Besides, nutrient status is an environmental factor that induces UPR in cancer cells. In fact, excess nutrients (fatty acids, cholesterol, and glucose) in the circulation observed in obesity and type 2 diabetes induces ER stress and UPR activation [57, 58]. Therefore, it will be of no surprise that UPR is activated in tumors from HFHS diet-fed mice. Herein, we observed an increase in the expression of UPR-related genes such as *Bip*, *Atf4*, and *Chop*, as well as an increase in the spliced form of *Xbp1* (Fig. 4A–D). Chop expression is related to the cell senescence process [58], consistent with the view that the Western diet is a key determinant of aging. Furthermore, we evaluated the expression of PERK, ATF6, and IRE1α proteins in tumors. Our results indicate an increase in PERK expression and phosphorylation at Thr981 (Fig. 4E, F), as well as in ATF6 (Fig. 4E,G) and IRE1α expression (1.88 times, Fig. 4E,H) in tumors from HFHS diet-fed group, as compared to chow-fed mice. In addition, it is also known that activated IRE1α cleaves *Xbp1* mRNA by removing the 26-base intron to produce the active form of *Xbp1s*, which regulates the transcription of target genes [59], supporting the data shown in Fig. 4E, H.

**Fig. 5:**
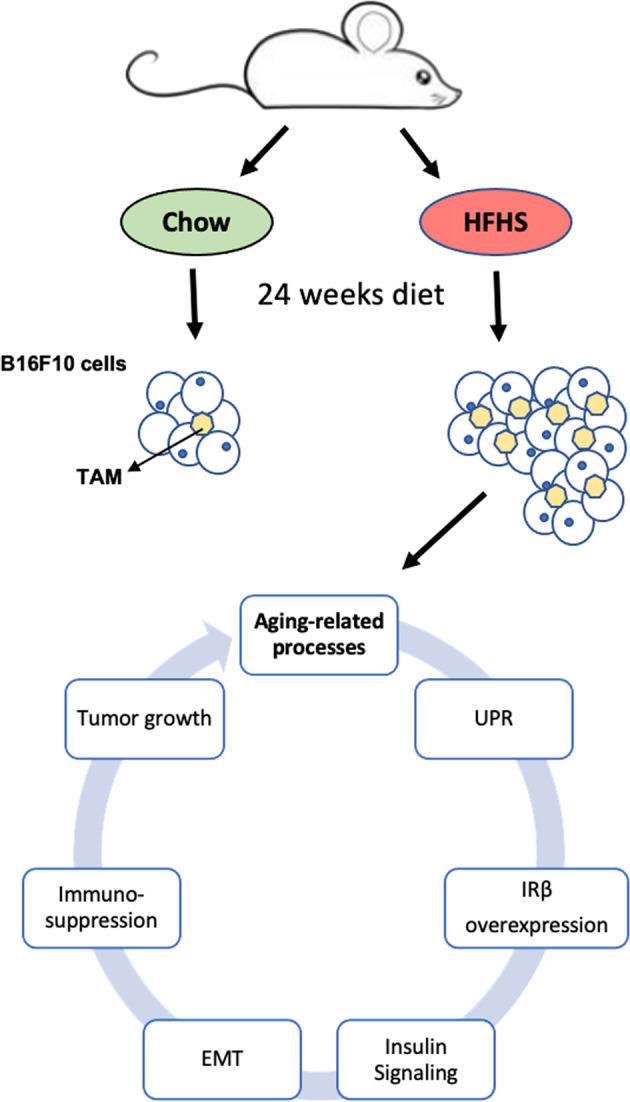
Summary of the effects of HFHS diet on tumor biology and inflammation. HFHS high-fat high-sucrose, TAM tumor-associated macrophage, UPR unfolded protein response, IRβ insulin receptor β, EMT epithelial–mesenchymal transition.

UPR activation, as previously described, is a key mechanism triggered by the HFHS diet, leading to the modulation of several cancer hallmarks. Of interest, it is now well established that angiogenesis is stimulated by ER stress and UPR activation in tumor and endothelial cells [60], since ATF4 and XBP1s directly bind to *Vegfa* promoter, which leads to an increase in *Vegfa* transcription. Moreover, HIF1α increases the stability of some UPR components, such as ATF4 and XBP1, contributing to the survival and chemoresistance of tumor cells [61, 62]. In addition, during malignant progression, tumor cells activate pathways that coopt cells in the tumor microenvironment, such as immune cells and endothelial cells, to support tumor growth. This scenario can involve the UPR pathway and this is accompanied by increased folding and secretion of cytokines, metalloproteinases, angiogenic factors, and components of the extracellular matrix [60, 63]. Hence, we conclude that the Western diet accelerates aging processes in mice, favoring tumor growth by stimulating M2-like macrophage (TAM) infiltration, activating the UPR pathway, and promoting EMT (Fig. 5), which collectively contribute to a poor prognosis.

## Conclusions

In summary, we have assessed the effects of an HFHS diet on tumor biology. We found an unexpected increase in tumor insulin sensitivity in the HFHS regimen, leading to enhanced tumor growth, and this was accompanied by the upregulation of genes involved in EMT, angiogenesis, and metastasis. However, other studies have already shown that overexpression of IR in cancer cells may explain their greater sensitivity to hyperinsulinemia. In addition, we observed an immunosuppressive response in tumors from HFHS diet-fed animals, which presented augmented macrophage infiltration with an M2-like polarization profile, which in turn contribute to tumor progression. Consistent with this physiological status, HFHS diet-induced UPR activation in tumors supports the diet-induced aging-related processes that drive tumorigenesis.

## Data Availability

The data used to support the findings of this study are available from the corresponding authors upon request.
